# Multifunctional applications for waste zinc–carbon battery to synthesize carbon dots and symmetrical solid-state supercapacitors†

**DOI:** 10.1039/d2ra00978a

**Published:** 2022-04-06

**Authors:** Phuoc-Anh Le, Van Qui Le, Nghia Trong Nguyen, Van-Truong Nguyen, Dang Van Thanh, Thi Viet Bac Phung

**Affiliations:** Institute of Sustainability Science, Vietnam Japan University, Vietnam National University Hanoi 100000 Vietnam lephuocanh86@vnu.edu.vn ptv.bac@vju.ac.vn; Department of Materials Science and Engineering, National Yang Ming Chiao Tung University Hsinchu 300093 Taiwan levanquidt@gmail.com; School of Chemical Engineering, Hanoi University of Science and Technology Hanoi 100000 Vietnam nghia.nguyentrong@hust.edu.vn; Faculty of Fundamental Sciences, Thai Nguyen University of Technology Thai Nguyen 24000 Vietnam vtnguyen@tnut.edu.vn; Faculty of Basic Sciences, Thai Nguyen University – University of Medicine and Pharmacy Thai Nguyen 24000 Vietnam thanhdv@tnmc.edu.vn

## Abstract

In this study, we provide a simple and green approach to recycle waste zinc carbon batteries for making carbon dots and porous carbon material. The carbon dots are easily synthesized by one green step, the hydrothermal treatment of a carbon rod in a mixture of DI water and pure ethanol to obtain a blue fluorescence under UV light, which can be used directly as a fluorescence ink. The as-prepared carbon dot process give typical dots with a uniform diameter from 3 to 8 nm with a strong slight blue fluorescent. The porous carbon material is also recycled from carbon powder in a waste battery *via* one green step annealing process without any chemical activation and with a hierarchically porous structure. This porous carbon material is demonstrated as an electrode for symmetrical solid state supercapacitors (SSCs) in a sandwich structure: porous carbon/PVA–KOH/porous carbon. The SSCs using recycled porous carbon electrodes exhibit a good energy density of 4.58 W h kg^−1^ at a power density of 375 W kg^−1^ and 97.6% retention after 2000 cycles. The facile one green step of hydrothermal and also that of calcination provide a promising strategy to recycle waste zinc carbon batteries, which transfers the excellent applications.

## Abstract 2

In this study, we provide a simple and green approach to recycle waste zinc carbon batteries for making carbon dots and porous carbon material.

## Graphical abstract

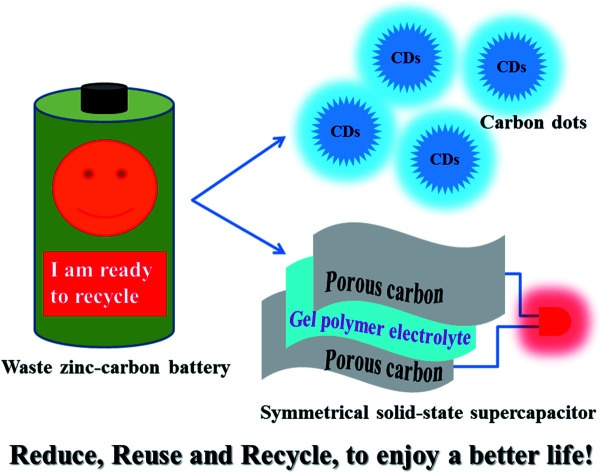

## 1 Introduction

Recently, energy storage is an interesting topic due to the necessity of energy in modern life. For a sustainable development in the future, the recycling of materials to protect the environment is always necessary.^1,2^ The development of energy storage devices depends on the economic ability to synthesize low-cost materials, abundant sources, and a stable quality.^3,4^ Carbon is a very common material that has appeared in human life for a long period of history as a source of energy. However, natural carbon supplies are limited so recycling to find other carbon sources is very important.^5^ Currently, scientists focus on carbon sources from the waste materials of industrial food, biomass, and recycled carbon sources, as they are abundant, cost-effective, and environmentally friendly.^6,7^ Moreover, with different biomass resources as the precursors we can obtain different structures of carbon materials.^8–10^

Nowadays, a huge amount of used batteries is discarded. This is the big environmental problem of every nation which faces a developing electronics industry. The zinc carbon battery is one of the oldest types but is still used everywhere, from TV remote controls, table clocks, to electronic equipment.^11,12^ All of the elements in a waste zinc carbon battery are not environmentally friendly so a suitable method to recycle them would truly useful. Herein, we provide a potential method to recycle the two main elements of a zinc carbon battery: the porous carbon powder and the carbon rod. The porous carbon contains a small amount of manganese dioxide, which is an ion transfer layer in the zinc carbon battery, but it is a dangerous industrial waste due to leaking waste materials and so a strategy is needed to recycle them. Moreover, the carbon rod in a zinc carbon battery works as a cathode and is a good raw material from which to synthesize carbon dots. Currently, with many outstanding properties, such as low toxicity, cost-effectiveness, easily dispersed in water, and high bio-adaptability, carbon dots have attracted more study in various fields, including optical technology (sensor, light-emitting diode), energy (catalyst, photovoltaics), and especially in biological applications (fluorescence *in vivo* bioimaging, nanomedicine).^13,14^

Supercapacitor research is attracted due to the high power density, fast charge–discharge, long lifetime and cycling stability.^15–17^ In this regard, various types of carbon materials, including commercial activated carbon, carbon nanotubes, graphite, graphene, biomass-derived porous carbon, industrial waste-derived activated carbon, have been studied as supercapacitor electrodes because of their advantages, such as versatile synthesis, high performance, and long-term stability.^18,19^ Many groups focus on developing supercapacitors using renewable sources, like biomass and industrial wastes, to synthesize carbon material as electrodes due to the low-cost, abundant sources, fast synthesis, high conductivity and large specific surface area.^20–23^ Different to previous reports, in this report, zinc carbon batteries were studied as industrial waste resources for the synthesis of carbon dots and porous carbon materials.

This report illustrates an excellent study to recycle waste zinc carbon batteries; one waste-resource for two applications. It demonstrates active carbons from recycling the zinc carbon battery for fluorescence ink and supercapacitors. Firstly, the carbon dots were synthesized *via* one hydrothermal step by using the carbon rods as a raw material. Secondly, we recycled composite carbon powder by one simple step calcination to obtain porous carbon material for electrodes in symmetrical solid state supercapacitors (SSCs).

## 2 Experimental

### 2.1 Material

The waste zinc carbon batteries were collected from recycled bins in supermarkets. Poly(vinyl alcohol) (PVA, 95% hydrolyzed, average *M*_w_ ∼ 95 000) was supplied from Acros. 1-Methyl-2-pyrrolidinone (C_5_H_9_NO) was purchased from Alfa Aesar. Carbon nanotube single wall, ethanol solution, potassium hydroxide (KOH), and poly(vinylidene fluoride) (PVDF, average *M*_w_ ∼ 534 000) were purchased from Sigma-Aldrich. The DI water was prepared with a Millipore Milli-Q UF system at room temperature.

### 2.2 Preparation of carbon dots

**Fig. 1:**
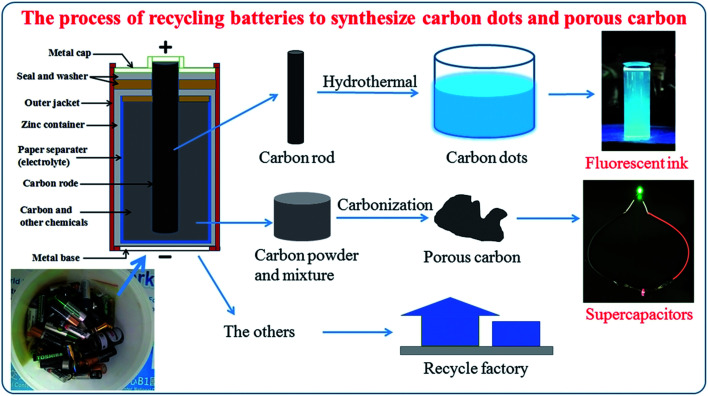
Process for the synthesis of carbon dots and porous carbon.

The carbon dots (CDs) were synthesized by a hydrothermal method (Fig. S1†). In the experiment, the carbon rod from the waste battery was ground to obtain carbon powder. 10 mg of this carbon powder was added to a 100 ml solution of DI water and ethanol (0.5 : 0.5). Then, this mixture was transferred into stainless steel autoclave, heated at 200 °C for 2 h and then cooled down to room temperature to obtain an opaque solution. The opaque solution was kept stable overnight and filtrated by a syringe filter 0.2 μm to remove large particles to form a CQD solution (Fig. 1).

### 2.3 Preparation of the porous carbon and symmetrical SSCs

Porous carbon was prepared from waste carbon battery (CB) powder by a one-step calcination method. In a typical preparation of porous carbon (Fig. 1 and S1†), 5 g of waste carbon battery powder was transferred into a ceramic crucible and annealed in an anaerobic furnace for 1 h at three different temperatures of 600 °C, named CB6, 800 °C, named CB8, and 1000 °C, named CB10. Then, the three resulting powders were filtrated, washed several times with DI water and ethanol, and dried at 80 °C overnight to obtain CB6, CB8 and CB10.

The carbon electrodes for symmetrical SSCs were made from four elements: 8 mg of active material (CB6, CB8 or CB10, 80 wt%), 1 mg of CNT (10 wt%), and 1 mg of PVDF (10 wt%) were mixed together in 0.2 ml NMP. These mixtures were stirred for 3 days to obtain homogeneous slurries. These slurries were coated on a carbon paper substrate over an area of 2 cm^2^ (1 × 3 cm, 1 cm^2^ for the current collector), then dried at 80 °C under a vacuum environment for 3 days to obtain porous carbon electrodes with 2 mg of each electrode.

The (PVA–KOH) gel polymer electrolyte (GPE) was synthesized by a solution casting method: 1 g of PVA and 1 g of KOH were mixed together in 20 ml of DI water with stirring at 80 °C for 2 h to obtain a homogeneous clear solution.

The symmetrical SSCs followed sandwich structure: CB6/PVA–KOH/CB6, named SCB6, CB8/PVA–KOH/CB8, named SCB8, CB10/PVA–KOH/CB10, named SCB10. Two electrodes were immersed in the electrolyte and dried in air. Then, one piece (1 × 2 cm) of oil absorbent paper was immersed in GPE and they were combined together under a high force and Scotch tape to obtain the devices (ESI video†).

### 2.4 Characterizations

The equipment used to study reusable porous carbon and carbon dots in this report were the following: photoluminescence spectroscopy (PL, PerkinElmer LAMBDA), ultraviolet-visible spectroscopy (UV-vis, F-7000 FL Spectrophotometer), X-ray diffraction spectroscopy (XRD, Bruker D2, Cu Kα tube), Raman spectroscopy (Jobin won, Horiba, Ar laser source with excitation wavelength of 520 nm), scanning electron microscopy (SEM, JEOL, JSM-6700F), transmission electron microscopy (TEM, JEM-ARM200F), X-ray photoelectron spectroscopy (XPS combined with Auger electron spectroscopy, Microlab 350), and the Brunauer–Emmett–Teller (BET) theory by porosity analyzer (Micromeritics, ASAP 2020).

In the electrochemical study, the symmetrical SSCs were tested by cyclic voltammetry (CV), electrochemical impedance spectroscopy (EIS) and galvanostatic charge–discharge (GCD) with the electrochemical workstation Zahner Zenium (Z 2.23, Germany) under room conditions.

The specific capacitance of the supercapacitor can be calculated *via* the CV curves by the equation:^24,25^1
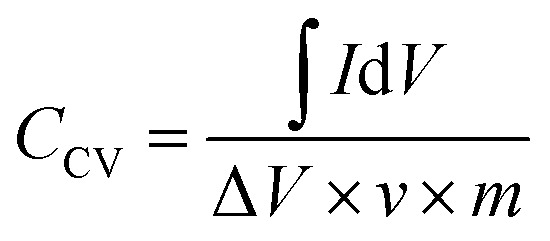
where *I* (A) is the current, Δ*V* (V) is the potential window, *ν* is the scan rate (mV s^−1^), and *m* (mg) is the mass of electrode layer.

The specific capacitance (*C*, F g^−1^) of the supercapacitor and symmetrical SSCs (*C*_s_, F g^−1^) were calculated using following equations:^24,25^2
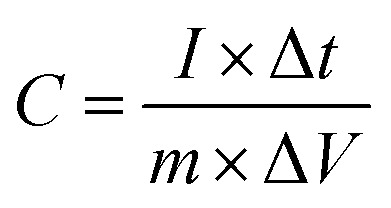

3 *C*_s_ = 4*C*

where Δ*t* (s) is the discharge time.

The energy density (*E*, W h kg^−1^) and the power density (W kg^−1^) were also calculated *via* the galvanostatic charge–discharge using these equations:^24,25^4
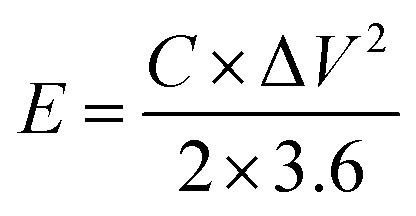
5
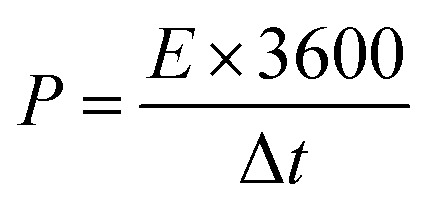


## 3 Results and discussion

Fig. 1 shows the general process to synthesize carbon quantum dots and porous carbon. One waste source for two applications (fluorescence ink and supercapacitors) provides a promising way to save natural sources and protect the environment.

### 3.1 Carbon dots

**Fig. 2:**
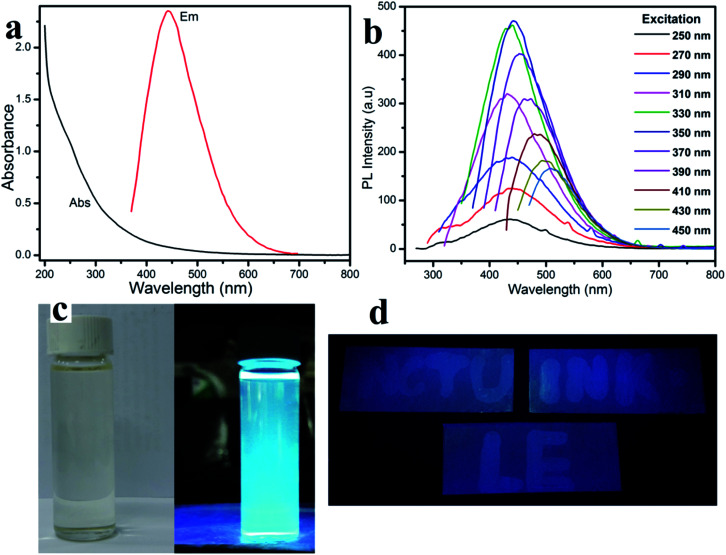
(a) UV-vis absorption spectra and emission spectra at an excitation wavelength of 350 nm, (b) fluorescent spectra of the CDs, (c) photograph of the CDs under daylight (left) and 365 nm UV light (right) and (d) photograph of QD ink on an aluminum thin-layer chromatography (TCL) plate.

**Fig. 3:**
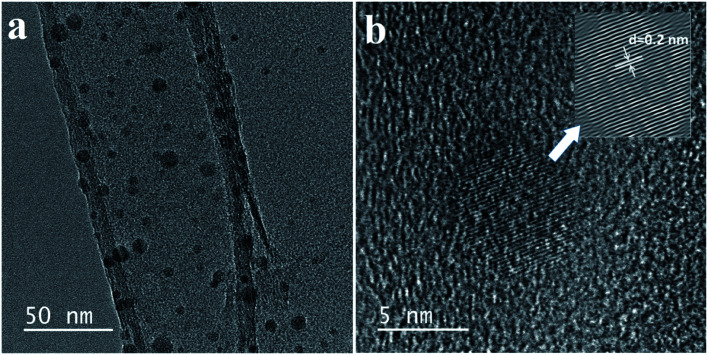
(a and b) TEM images of the carbon quantum dots.

The fluorescent property shown in Fig. 2a, displays the one peak of the CD absorbance at around 250 nm. Therefore, the CD solution demonstrates a strong fluorescence with an excitation wavelength from 250 nm to 450 nm (Table S1†). Especially, the photoluminescence (PL) intensity reaches a maximum at an excitation wavelength of around 450 nm, which agrees with the CD aqueous solution displaying a slightly blue fluorescence under UV light. In ambient conditions, the CD solution has excellent transparency, indicating a good distribution of the quantum dots (Fig. 3c-left),^26,27^ and the CD solution shows a bright blue fluorescent under a 365 nm UV light lamp (Fig. 3c-right).

Fig. 3 shows the morphological characterization of the carbon dots *via* TEM measurement. The TEM images clearly indicate that the carbon dots are dispersed homogeneously on the surface of the lacey carbon of the Cu grids. From Fig. 3a, the carbon dots have a good size distribution in diameter from 3 to 8 nm. At a high resolution (Fig. 3b), the clearly lattice fringes indicate that almost all of the carbon dots have a crystal structure with a lattice spacing of approximately 0.2 nm.^26–29^

**Fig. 4:**
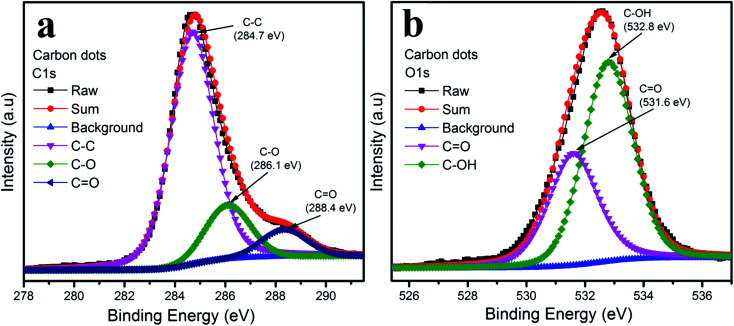
XPS spectra of carbon dots: (a) C 1s and (b) O 1s.

The functional groups on the surface of the carbon dots were examined by XPS measurement shown in Fig. 4. The XPS spectra of the carbon dots were attributed to carbon and oxygen. The XPS spectra of C 1s in Fig. 4a could be deconvoluted into three peaks: graphitic sp^2^ carbon C–C (284.7 eV), C–O (286.1 eV) and C=O (288.4 eV).^30–33^ As depicted in Fig. 4b, the O 1s spectrum illustrates two peaks at 531.6 eV and 532.8 eV, which are attributed to C=O and C–OH, respectively.^30^

In conclusion, one facile step to synthesize carbon dots from the carbon rods in a waste battery was investigated carefully. All of the above results indicate a good quality of carbon dots and excellent potential in fluorescence applications, such as fluorescence ink.

### 3.2 Waste carbon battery-derived porous carbon material

**Fig. 5:**
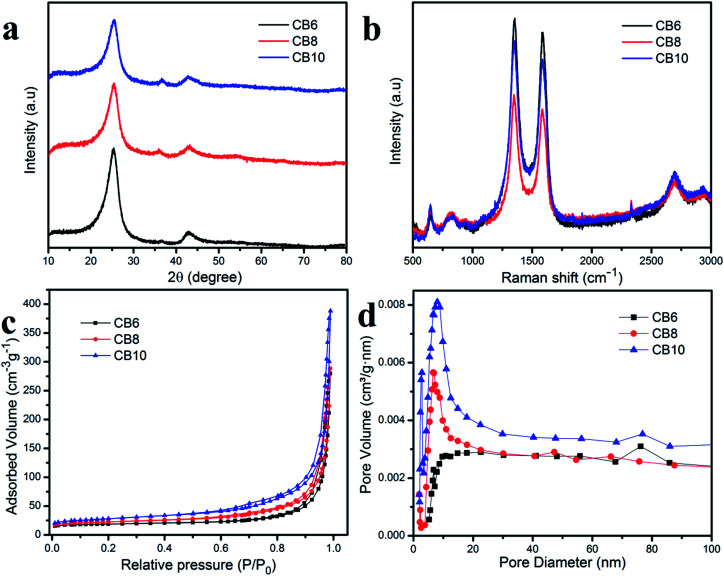
(a) XRD pattern, (b) Raman spectra and (c and d) BET measurements of porous carbon powders.

Fig. 5a shows the wide angle XRD pattern of these porous carbon materials at the various different calcination temperatures. The high peak at 2*θ* of about 25° was assigned to (002) and the small broad peak at 2*θ* of about 43.8° was attributed to (001), which correspond to graphitic carbon and a disordered structure.^34,35^ Moreover, the high intensity of the peak at 2*θ* = 25° indicates a high concentration of the crystalline phase in the porous carbon materials which is consistent with the TEM results.^36^ Furthermore, the clearly broad peak at 2*θ* = 43.8° demonstrates the condensation of carbon material, which helps to improve the electrical conductivity.^37,38^ Raman scattering measurements were used to investigate further the structure of the porous carbon materials. In Fig. 5b, the porous carbon materials CB6, CB8 and CB10 show two clear peaks located at about 1345.5 (D band) and 1585.3 (G band). The D band is related to the disordered graphite structure defect and the G band corresponded to the 2D graphite lattice vibrational mode.^38–40^ The relative intensity between the D band and G band (*I*_D_/*I*_G_) reflects the crystallinity of these porous carbon materials. In this condition, the ratio *I*_D_/*I*_G_ = 10.4, 1.06 and 1.05 for CB6, CB8 and CB10, respectively, which indicates the high degree of graphitization and low degree of disordered structure. These results demonstrate a good conductivity due to the high crystallinity phase in porous carbon materials.^36–40^

The nitrogen adsorption–desorption isotherm measurement was examined for information about the specific surface area and pore structure of porous carbon materials and is depicted in Fig. 5c and d. It can be seen that the specific surface areas of the porous carbon materials at different calcination temperatures increase slightly and the pore size distribution is quite uniform. These results indicate stable and uniform porous carbon materials after recycling from the carbon of waste batteries. The specific capacitances of CB6, CB8 and CB10 are 64.3, 78.1 and 97.6 m^2^ g^−1^, respectively. The pore size of the porous carbon materials is mainly distributed in the range between 20 and 30 nm, which demonstrates a suitable size as an electrode for energy storage applications.

**Fig. 6:**
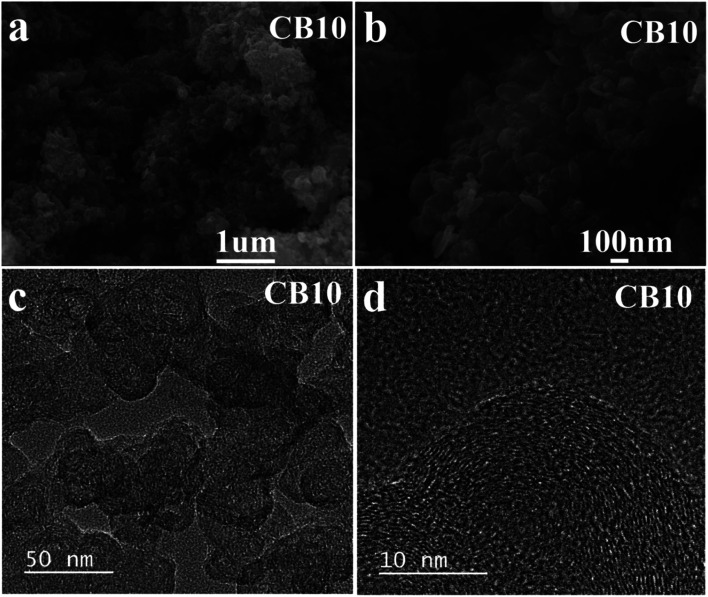
(a and b) SEM and (c and d) TEM images of porous carbon CB10 at various magnifications.

The SEM images of porous carbon material CB10 are shown in Fig. 6a and b, which exhibits a uniform spherical structure. The spherical structure of the carbon materials with a high crystalline phase in a disordered distribution is a factor that improves the conductivity and an excellent choice for electrode materials.^41,42^ Moreover, high resolution SEM of CB6, CB8, and CB10 is also illustrated in Fig. S3† at various magnification, and the uniform spherical structure shown clearly with diameters from 20 to 30 nm with a porous structure. Furthermore, the TEM images of CB10 in Fig. 6c and d show more detail about porous structure with a high crystallinity phase. At low resolution (Fig. 6c), the porous carbon material confirms a uniform spherical structure with a disordered distribution. At high resolution TEM (Fig. 6d), the overlap of some thin layers can be clearly seen, which makes the porous carbon structure have a large fraction of micro pores with clear lattice fringes in the particle, which is from the graphitic carbon matrix. Thus, this structure can improve the conductivity of this porous carbon material as a potential candidate for supercapacitor application.

**Fig. 7:**
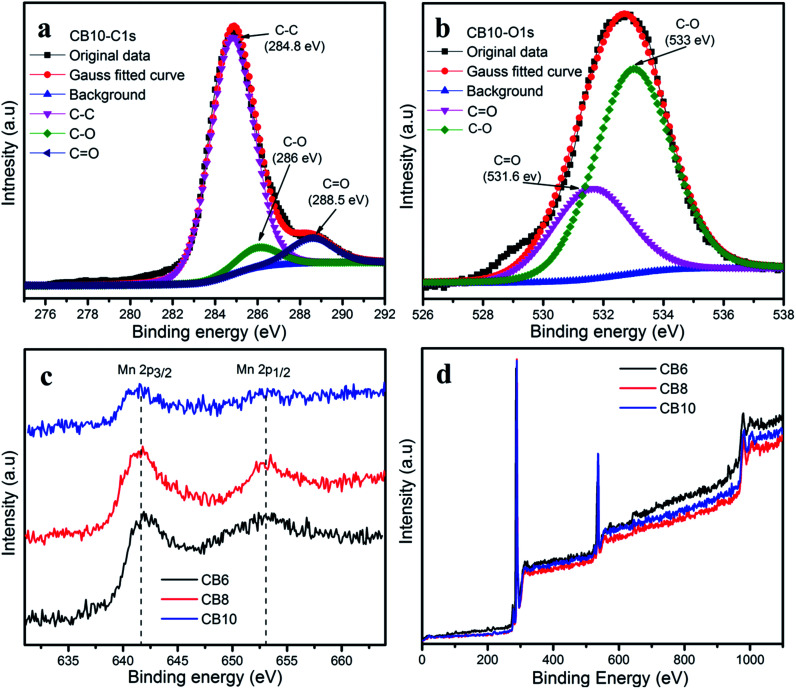
(a) C 1s and (b) O 1s XPS spectra of CB10. (c) Mn 2p XPS spectra and (d) survey spectra of CB6, CB8 and CB10.

The species and chemical composition of the porous carbon materials were determined by XPS measurements and depicted in Fig. 7 and S2.† The survey spectra (Fig. 7d) show two clear peaks, which correspond to C 1s and O 1s. As can be seen, Fig. 7a shows the C 1s spectra of CB10 with three peaks at 284.8, 286 and 288.5 eV, which are attributed to the C–C bonds, C–O bonds and C=O bonds.^37,39,40^ The O 1s spectrum of CB10 is deconvoluted into two peaks at 531.6 and 533 eV, which correspond to the C=O and C–O bonds.^37,40^ The existence of the abundant oxygen in the chemical groups can increase the active surface area and pseudocapacitance to enhance the total capacitance of a supercapacitor.^38^ In the survey spectra, the existence of the manganese element is very weak and the Mn 2p core level spectrum is shown in Fig. 7c. The Mn 2p illustrates the two broad weak peaks of Mn 2p_3/2_ and Mn 2p_1/2_ are attributed at 642.3 eV and 653.8 eV, with a spin orbit splitting of 11.5 eV. This result agrees with those reported for the XPS profile of MnO_2_.^41–44^

### 3.3 Symmetrical SSCs devices

**Fig. 8:**
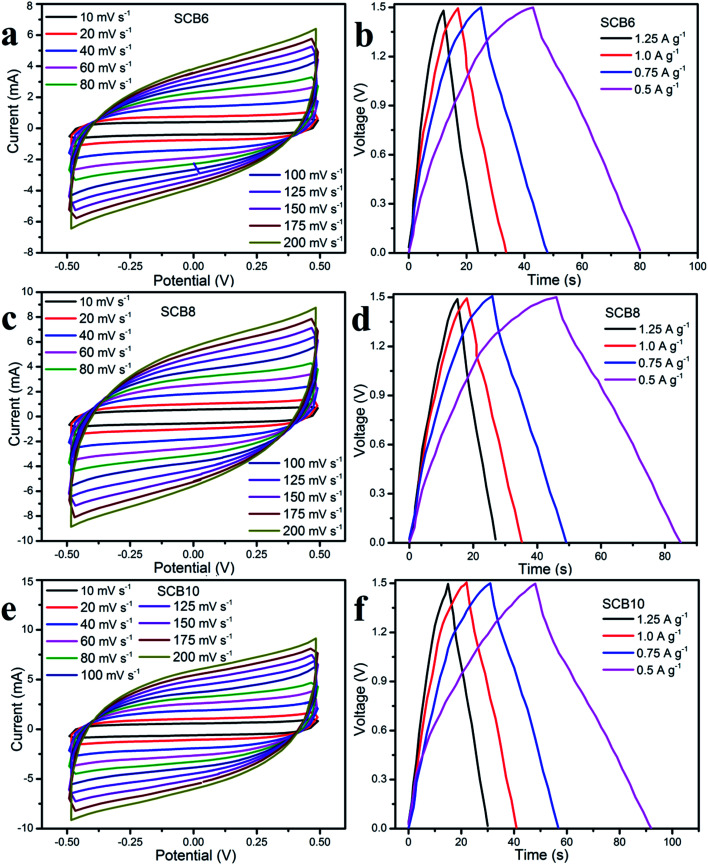
Electrochemical properties of the SSCs of SCB6, SCB8 and SCB10: (a, c and d) CV curves and (b, e and f) GCD curves, respectively.

In order to investigate the electrochemical performances of the porous carbon materials, three symmetrical solid-state supercapacitors were assembled for characterization. Fig. 8 shows the CV curves and GCD curves of SCB6, SCB8 and SCB10 at various scan rates and current densities. The CV curves of SCB6, SCB8 and SCB10 at various scan rates from 10 to 100 mV s^−1^ have a rectangular shape with our redox peaks, indicating an excellent capacitive behavior of the electrochemical double layer mechanism.^34,45,46^ All GCD curves [Fig. 8b, d and f] illustrate the quasi-triangular shape, indicating an efficient ion transport and ideal electrical double layer capacitor behavior.^47^ Moreover, at various current densities from 0.5 to 1.25 A g^−1^, the GCD curves show linearity with an excellent charge–discharge invertibility of porous carbon electrodes.^46^

**Fig. 9:**
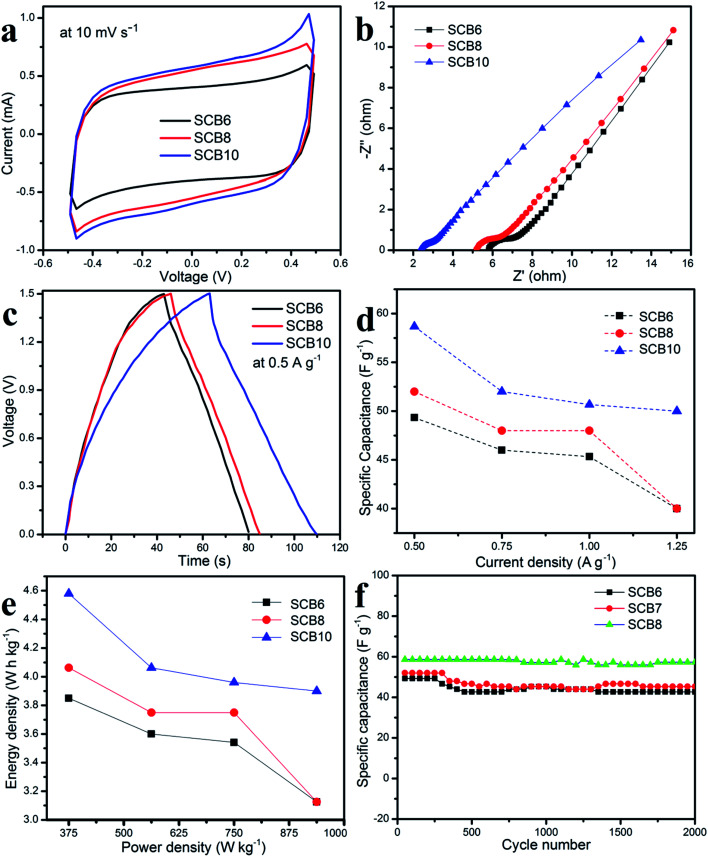
Electrochemical properties of the symmetrical SSCs: (a) CV curves, (b) EIS plots, (c) GCD curves, (d) specific capacitance as a function of current density, (e) Ragone plots and (f) cycling stability.

As shown in Fig. 9a, it can be clearly seen that SCB10 has a bigger CV curve than those of SCB6 and SCB8 at the same scan rate of 10 mV s^−1^, indicating the higher specific capacitance of SCB10. From eqn (1), the specific capacitance of the symmetrical SSCs (*C*_CV_) of SCB6, SCB10 and SCB10 are 39.85, 52.3 and 58 F g^−1^ at 10 mV s^−1^, respectively. To further investigate the ion transport and internal impedance of the symmetrical SSCs, EIS measurements were studied. Fig. 9b shows the EIS results *via* Nyquist plots of SCB6, SCB8 and SCB10 in the range 100 mHz–100 kHz with an amplitude of 5 mV. The linear slopes of the Nyquist plots in the low-frequency region confirm the mass transfer process, which indicates its faster ion diffusion in an electrolyte.^47,48^ The low equivalent series resistances (ESR) of the EIS measurements of the supercapacitors illustrate the good conductivity and capability of the supercapacitor cells.^49,50^ The equivalent series resistances of SCB6, SCB8 and SCB10 are 1.94, 1.5 and 1.25 Ω, respectively. Here, the ESR includes the resistance of the interface electrode/electrolyte, the intrinsic resistance of electrode and the resistance of the KOH electrolyte.^30^ The charge–discharge profiles in Fig. 9c show the same quasi-triangular shape for all CB6, CB8 and CB10 electrode materials, confirming that the porous carbon materials have a stable structure despite carbonization to different degrees. Moreover, from Fig. 9c, the discharge time of SCB10 is longer than those of SCB6 and SCB8, indicating a higher specific capacitance. To investigate the electrochemical performances of the supercapacitor devices, the specific capacitances of the supercapacitors at various current densities were calculated *via* GCD curves based on eqn(2) and (3), and shown in Fig. 9d. The maximum specific capacitance of symmetrical SSC cells using the SCB6, SCB8 and SCB10 electrodes were 49.3, 52 and 58.7 F g^−1^, respectively, at a current density of 0.5 A g^−1^. Fig. 9e shows the relation between the energy density and power density of a symmetrical SSC cells using SCB6, SCB8 and SCB10 *via* Ragone plots which can be calculated from the GCD profiles. The maximum energy density of SCB10 is 4.58 W h kg^−1^, which is higher than those of SCB6 (3.85 W h kg^−1^) and SCB8 (4 W h kg^−1^) at a power density of 375 W kg^−1^. Even when the maximum power density is 937.5 W kg^−1^, the energy density of SCB10 is 3.9 W h kg^−1^ which is higher than those of SCB6 (3.1 W h kg^−1^) and SCB8 (3.125 W h kg^−1^). Further, the cyclic stability of the symmetrical SSCs reflects the energy storage performances which were investigated at a current density of 0.5 A g^−1^ after 2000 cycles and are illustrated in Fig. 9f. After 2000 cycles, the specific capacitance of the symmetrical SSC device of SCB10 retains about 97.6% of the initial capacitance which is higher than those of SCB6 (86.6%) and SCB8 (87.2%) (Table 1). Furthermore, the series of two symmetrical SSCs could run a blue LED, which indicates the potential device application (ESI video†). In conclusion, the symmetrical SSCs using porous carbon electrodes (CB6, CB8 and CB10) can be considered as good candidates for high-performance solid-state supercapacitors in the comparison with other waste resources (Table 2).

**Table 1:** Electrochemical performance of SSCs in this report

Samples	Specific surface area (m^2^ g^−1^)	Electrochemical performance		
		Specific capacitance (F g^−1^)	Maximum energy density (W h kg^−1^)	Capacitance retention after 2000 cycles
CB6	64.3	49.3 F g^−1^ at 0.5 A g^−1^	3.85	86.6%
CB8	78.1	52 F g^−1^ at 0.5 A g^−1^	4	87.2%
CB10	97.6	58.7 F g^−1^ at 0.5 A g^−1^	4.58	97.6%

**Table 2:** Comparison of waste zinc carbon battery-derived porous carbon with other waste resource-derived carbon materials for solid-state supercapacitors

Precursor	Activation agent	Method	Electrolyte	Electrochemical performance	Ref.
Bamboo char	K_2_FePO_4_	Carbonization	PVA–KOH	48.1 F g^−1^ at 0.2 A g^−1^	15
Areca palm leaves	KOH	Carbonization	PVA–Li_2_SO_4_	132 F g^−1^ at 0.5 A g^−1^	17
Carbon wood/PANI	Anilin/HCl/(NH_4_)_2_S_2_O_8_	Carbonization & polymerization	PVA–LiOH	27 mF cm^−2^ at 0.5 mA cm^−2^	20
Waste oily sludge	KOH	Carbonization	PVA–KOH	81.3 F g^−1^ at 0.5 A g^−1^	51
Pitch	*N*,*N*′-Diphenylthiourea & C_7_H_5_KO_2_	Polymerization& carbonization	PVA–Na_2_SO_4_	61 F cm^−3^ at 0.3 A cm^−3^	52
Waste zinc–carbon batteries	**Without using**	Carbonization	PVA–KOH	58.7 F g^−1^ at 0.5 A g^−1^	**This work**

## 4 Conclusions

In summary, the carbon dots and porous carbon materials were synthetized by a simple method with a high quality for potential applications in fluorescence ink and energy storage. The best advantage of this report is its environmental method to recycle used batteries: the carbon dots were synthesized in a mixture of ethanol and water, and the porous carbon was calcinated without any chemical activation. The carbon dots have a strong fluorescence under UV light which can be used for fluorescence ink application. The porous carbon materials have a high conductivity and good specific surface area. For supercapacitor applications, the porous carbons (CB6, CB7 and CB8) showed a good electrochemical performance. The maximum specific capacitance of the symmetrical solid-state supercapacitor devices for SCB6, SCB8 and SCB10 were 49.3, 52 and 58.7 F g^−1^ at a current density of 0.5 A g^−1^. Moreover, the devices showed a long cycle stability, especially the specific capacitance retained at about 97.6% after 2000 cycles for SCB10. The carbon dots and porous carbon in this report bring a potential method to recycle waste batteries and so protect the environment.

## Author contributions

The manuscript was written through contributions by all the authors. All the authors have given approval to the final version of the manuscript.

## Conflicts of interest

The authors declare no competing financial interest.

## Supplementary Material

RA-012-D2RA00978A-s001

RA-012-D2RA00978A-s002
