# Supplementary material for: Multifunctional applications for waste zinc–carbon battery to synthesize carbon dots and symmetrical solid-state supercapacitors
Source: RSC Adv. 2022 Apr 6;12(17):10608–18. doi: 10.1039/d2ra00978a (PMC8984403; doi:10.1039/d2ra00978a)
Supplement: RA-012-D2RA00978A-s001 [file RA-012-D2RA00978A-s001.pdf]

Electronic Supplementary Material (ESI) for RSC Advances.
This journal is © The Royal Society of Chemistry 2022

Supporting information

Multifunctional applications for waste zinc-carbon battery to synthesize carbon dots and symmetrical solid-state supercapacitors

Phuoc-Anh Le[1]*, Van Qui Le[2], Van-Truong Nguyen[4], Dang Van Thanh[5], Nghia Trong Nguyen[3], Thi Viet Bac Phung[1]*

[1] Institute of Sustainability Science, Vietnam Japan University, Vietnam National University, Hanoi 100000, Vietnam

[2] Department of Materials Science and Engineering, National Yang Ming Chiao Tung University, Hsinchu 300093, Taiwan

[3] School of Chemical Engineering, Hanoi University of Science and Technology, Hanoi 100000, Vietnam.

[4] Faculty of Fundamental Sciences, Thai Nguyen University of Technology, Thai Nguyen 24000, Viet Nam.

[5] Faculty of Basic Sciences, Thai Nguyen University - University of Medicine and Pharmacy, Thai Nguyen 24000, Viet Nam

***Corresponding author:**

Phuoc-Anh Le; E-mail: lephuocanh86@vnu.edu.vn

Thi Viet Bac Phung; E-mail: ptv.bac@vju.ac.vn

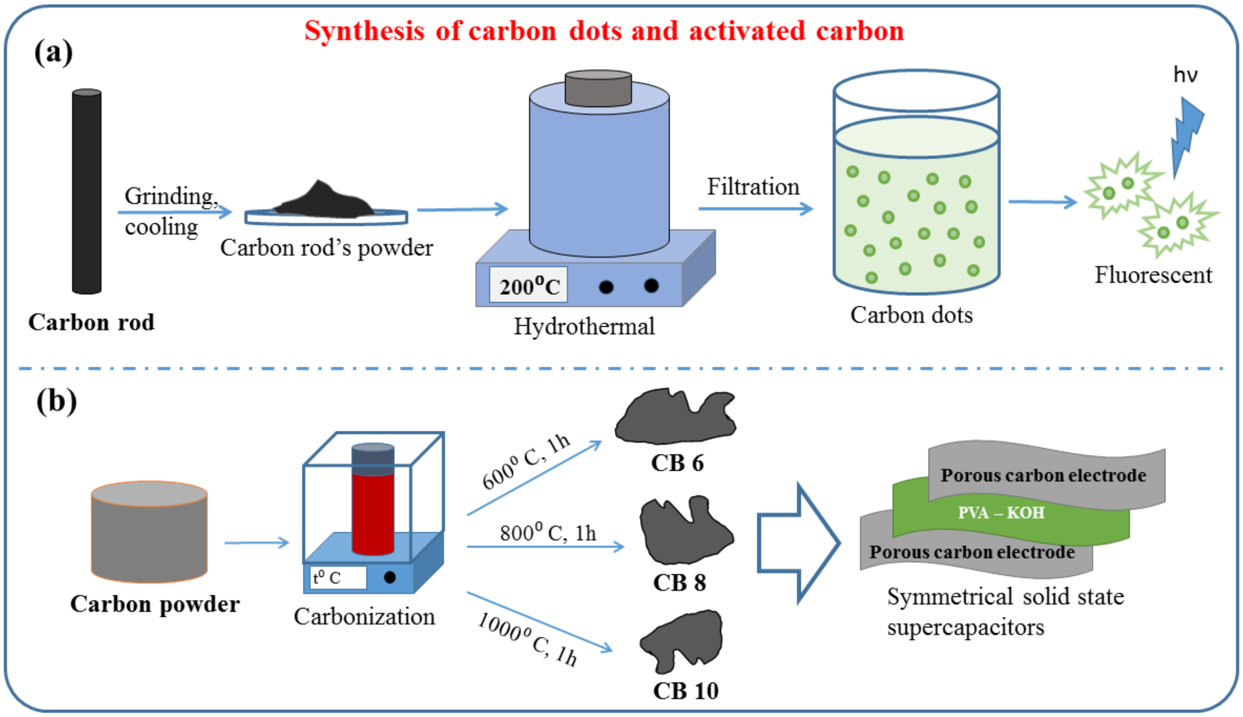

Figure S1 The detail schematic for preparation of carbon dots and porous carbon materials.

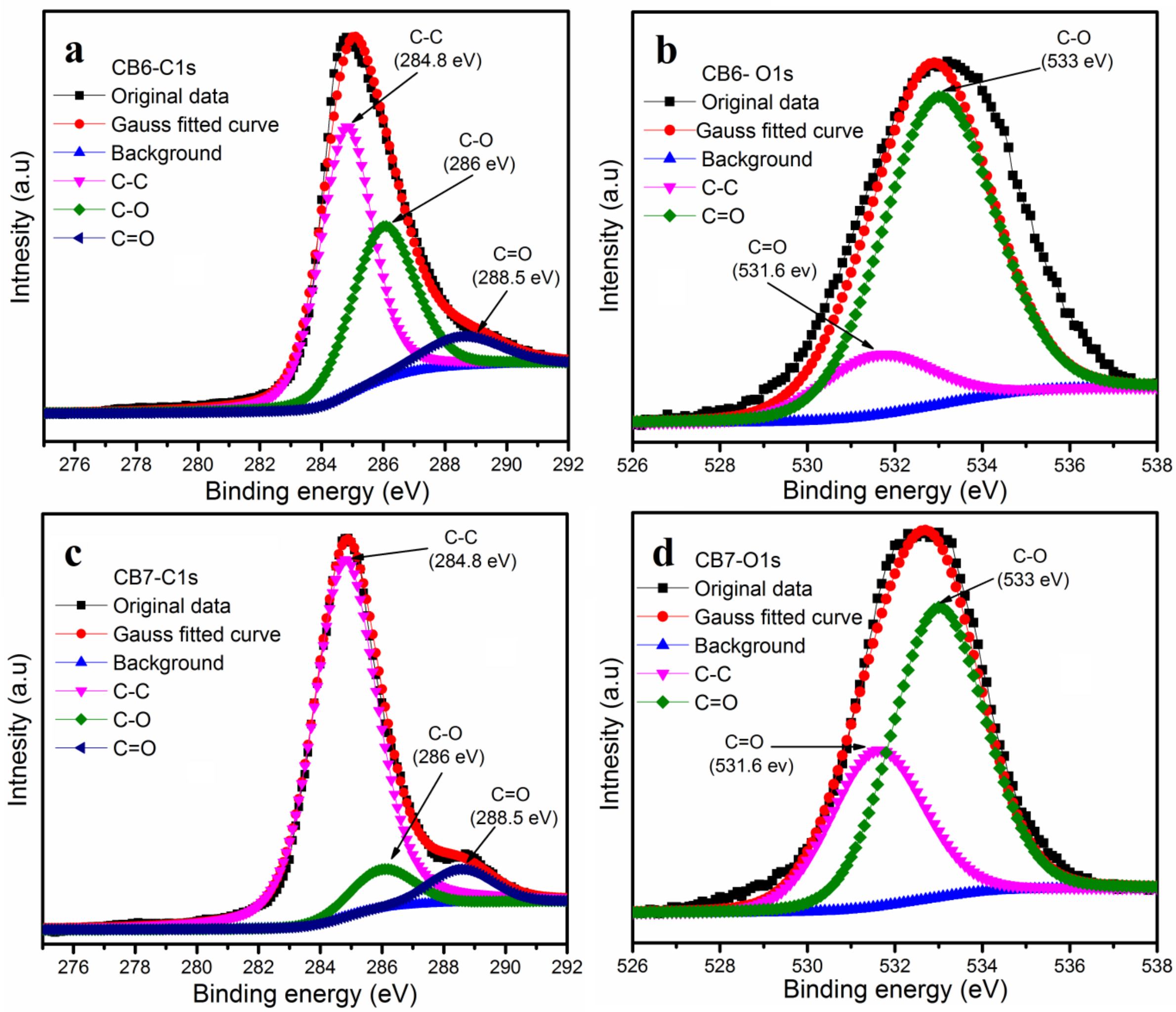

Figure S2 XPS spectra C1s and O1s of CB6 and CB8.

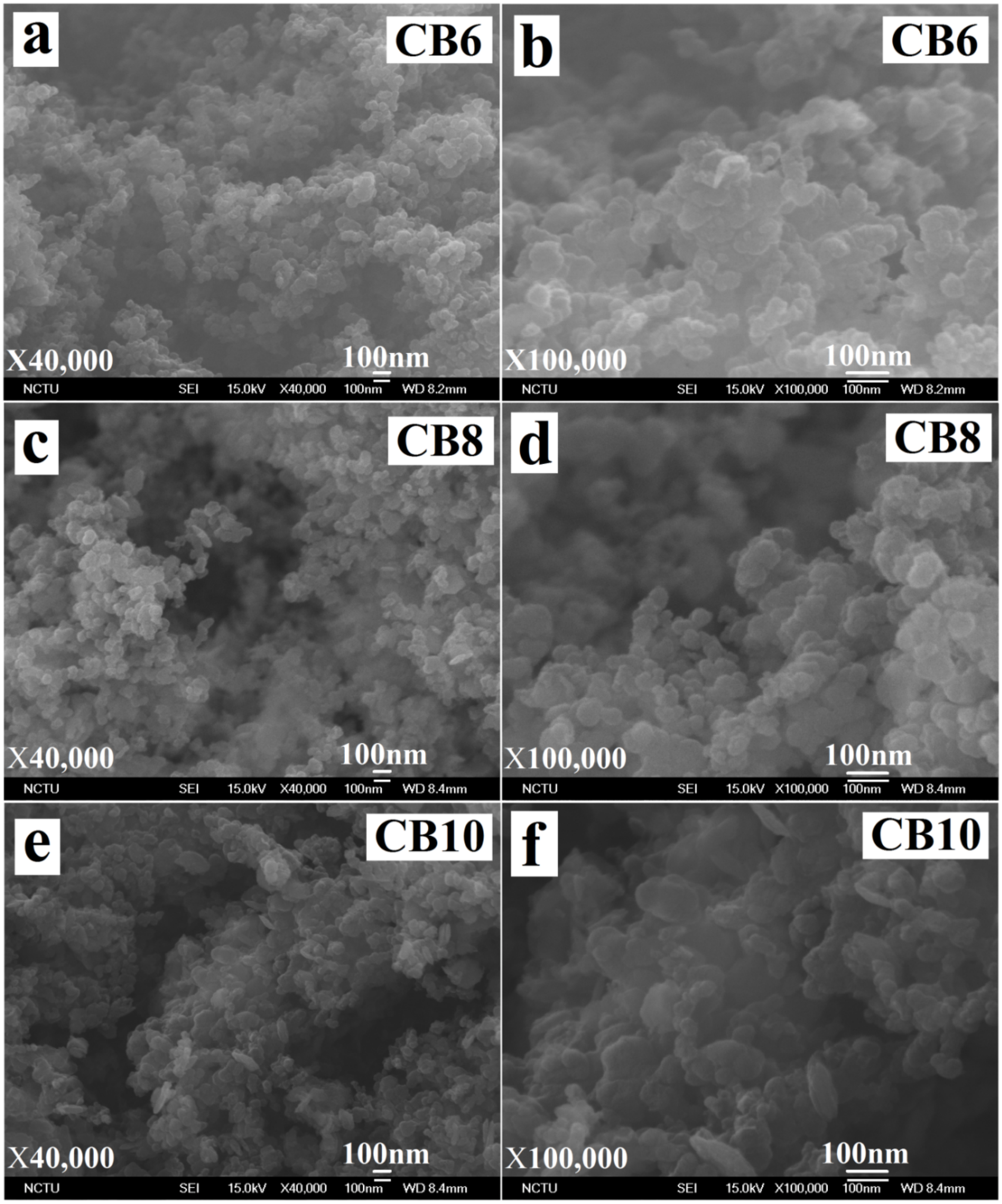

Figure S3 SEM images of (a, b) CB6, (c, d) CB7 and (e, f) CB8 at high magnification.

Table S1 Detail table of PL excitation following UV-Vis data

UV-Vis absorption of 250 nm											
PL excitation (nm)	250	270	290	310	330	350	370	390	410	430	450
PL peak (nm)	435	440	435	430	435	450	455	470	485	500	510
